# Schwannoma (neurilemmoma) of tongue: A rare case presentation and review of literature

**DOI:** 10.1002/ccr3.7235

**Published:** 2023-04-22

**Authors:** Mahboobe Asadi, Malihe Mohseni, Fatemeh Jahanshahi, Arash Esmaeili, Zhaleh Mohsenifar

**Affiliations:** ^1^ Otorhinolaryngology and Head and Neck Surgery Department, Taleghani Hospital Shahid Beheshti University of Medical Sciences Tehran Iran; ^2^ Research Committee, Faculty of Medicine Iran University of Medical Sciences Tehran Iran; ^3^ Department of Pathology, Taleghani Hospital Shahid Beheshti University of Medical Sciences Tehran Iran

**Keywords:** oral cavity, schwannoma, tongue schwannoma

## Abstract

It seems that schwannomas of the tongue base originate from branches of the glossopharyngeal, vagus, or hypoglossal nerves. Additionally, complete trans‐oral surgical excision is an efficient method to remove them.

## Abstract 2

Tongue Schwannoma is rare, and a definite diagnosis is made only with histopathological examination. In the current study, we present a case of tongue schwannoma in a 21‐ year‐old male case, with radiological and histopathological investigations.

## 1 INTRODUCTION

Schwannomas (Neurilemmomas) are an encapsulated and generally benign tumors that originate from Schwann cells around peripheral nerve fibers.^1^ It has been reported that about a quarter to a half of Schwannomas are found in the head and neck regions,^2^ and about 1%–12 % of them are found in the oral cavity.^3^ However, the tongue Schwannoma has a very low prevalence, and few cases have been reported.^4^, ^5^, ^6^ Though the precise etiology of Schwannoma is unknown, some relevant risk factors like chronic irritation, external injury, stress oxidative, exposure to radiation, or genetic susceptibility have been reported in literatures.^7^ Schwannomas have a same‐gender preference, and the age range of thirty to fifty years is more likely for its occurrence.^8^ Tongue schwannoma is most commonly observed in the 2nd to fourth decades of life,^9^ moreover some studies have reported its prevalence in women (52.8%) and men (47.2%), which indicating that the role of sex is not important in its pathogenesis.^10^ In addition, some systematic review studies have reported that about 70% of tongue schwannomas are painless.^10^ Complete resection of the mass via trans‐oral approach is the priority treatment, and the tumor recurrence chances after surgery are low.^11^ In the current study, we present a case of tongue schwannoma in a 21‐year‐old male case, with radiological and histopathological investigations.

## 2 CASE REPORT

**FIGURE 1:**
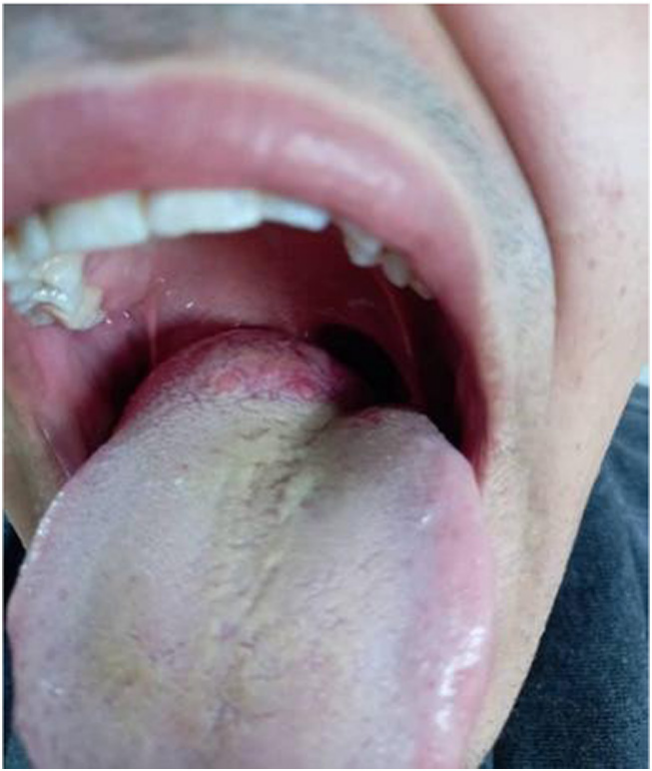
The patient's base of tongue image.

The patient is a 21‐year‐old man with a lump on the right side of his tongue which was observed during a dental examination. For more detailed evaluations, the patient was referred to the ENT ward of Taleghani Hospital of Shahid Beheshti University of Medical Sciences, Tehran, Iran. The patient's chief complaint was swelling of the base of the tongue (Figure 1), which had worsened 2 weeks earlier. The patient was serving in the military. There was no positive history of dysphasia, odynophagia, snoring, drooling, feeling of asphyxia, fever and night sweats, obstructive sleep apnea, abnormal weight loss, pain, bleeding, taste disturbances, and post throat discharge. The patient had no history of surgery on head and neck areas such as tonsillectomy, frenectomy, skull base surgery, etc. However, the patient had underwent a surgery for a broken tibia 8 years ago.

Furthermore, his family history was not remarkable. On intraoral evaluations, the general and special sensation of the tongue, the sensation of buccal mucosa, and mandibular movements were normal, and no signs of abscess or ulcer were seen in the patient's mouth. Cervical lymph nodes were not palpable, and no sign of lymphadenopathy was also seen; the remaining clinical and Para clinical examination was normal.

**FIGURE 2:**
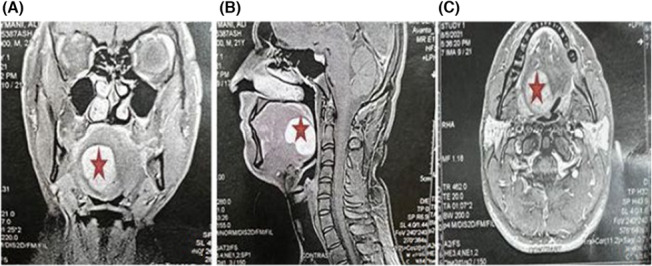
Soft tissue mass with irregular and smooth border in the right posterior aspect of tongue was seen on MRI sections. (A) Coronal view, (B) Sagittal view, (C) Axial view.

The magnetic resonance imaging (MRI) was requested for more evaluation. According to the MRI findings, soft tissue mass with irregular and smooth border in the right posterior aspect of the tongue measuring about42.5 × 40 × 26 mm mildly low on T1 weighted images (T1WI) and high on T2 weighted images (T2WI) with homogenous enhancement after IV Gd was seen (Figure 2).

**FIGURE 3:**
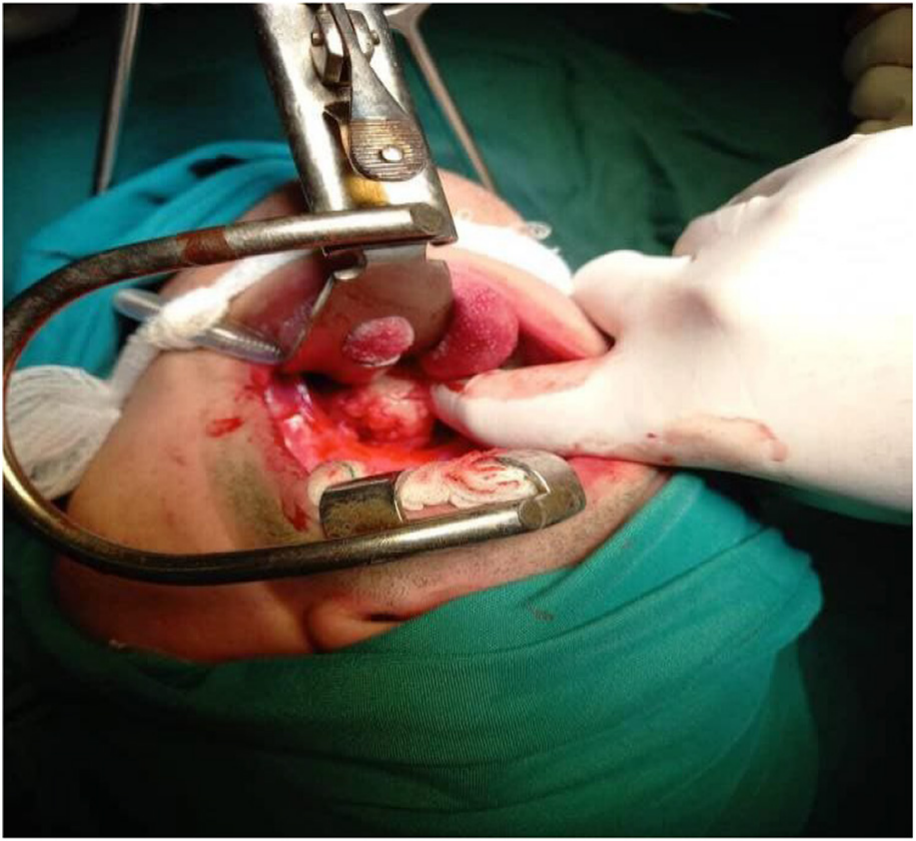
Complete transoral surgical excision.

**FIGURE 4:**
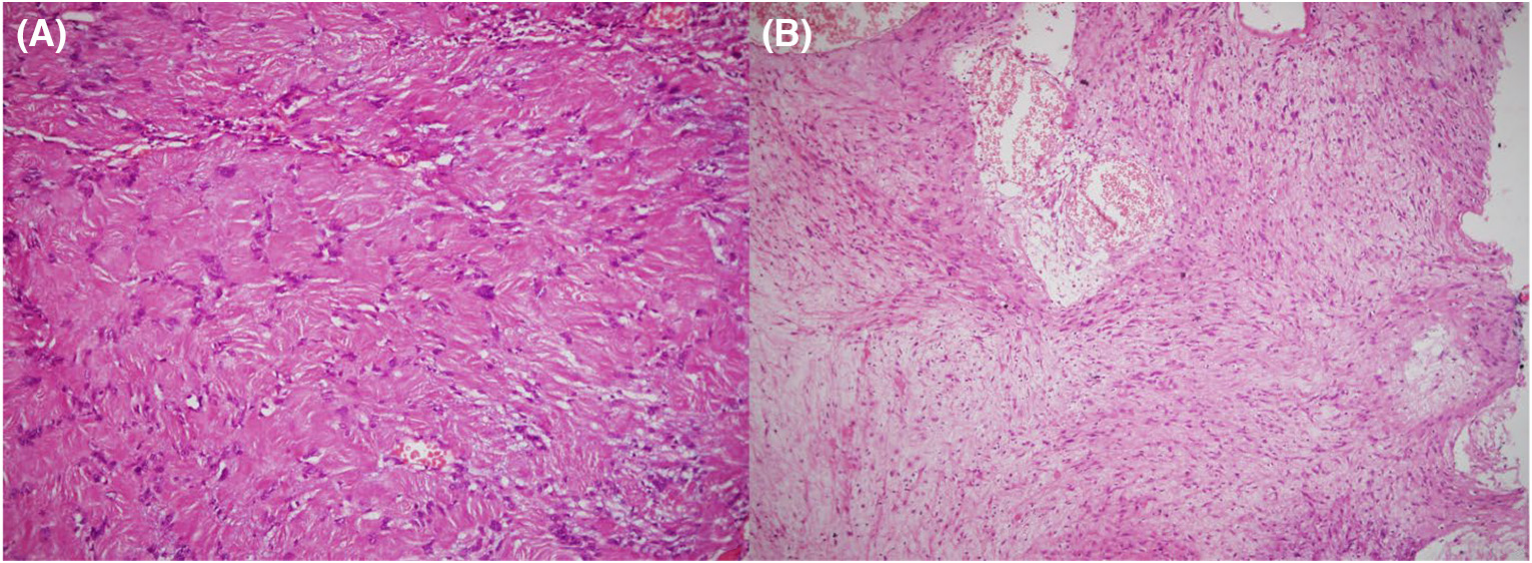
Sections show biphasic neoplasm (A) Hypercellular Antoni A area shows nuclear palisading and Verocay bodies. (B) Myxoid hypocellular Antoni B area. Nuclei are wavy and elongated.

**FIGURE 5:**
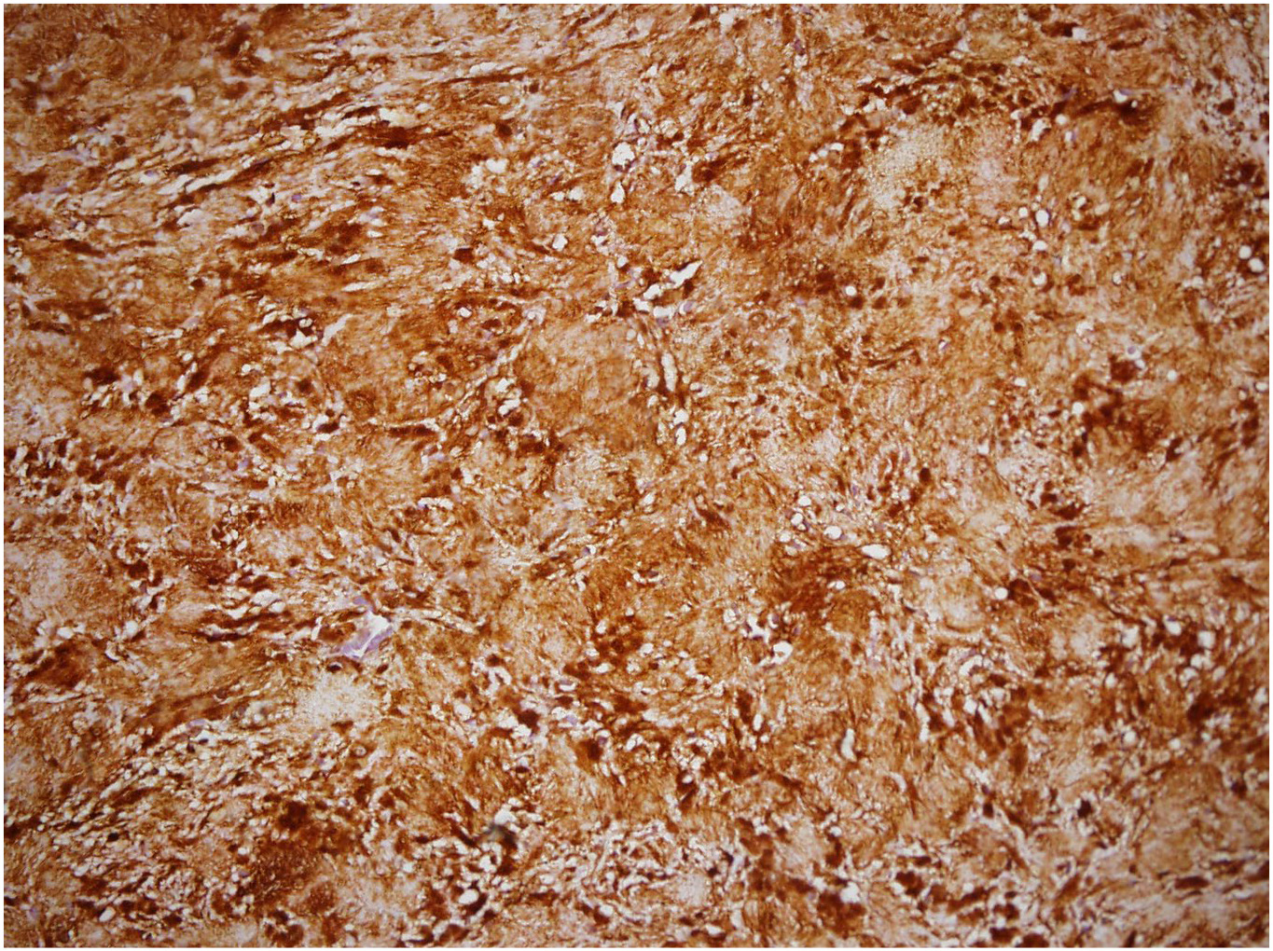
IHC staining for S100 is positive.

The patient's MRI showed no sign of a mass invasion of the surrounding tissues and appeared to be a benign mass such as schwannoma, neurofibroma, or leiomyoma. Complete transoral surgical excision was conducted under general anesthesia without complications (Figure 3). The removed mass was sent to the pathology department for a more detailed histopathological evaluation. According to the pathology report, the removed mass was compatible with schwannoma (Figure 4). Moreover Immunohistochemistry study was conducted. (Figure 5).

## 3 DISCUSSION

Schwannomas are benign tumors that result from the abnormal proliferation of Schwann cells, which are responsible for generating myelin.^1^ These neoplasms can arise from peripheral, autonomic, and cranial nerves (except olfactory and optic nerves).^1^, ^2^ Most of these schwannomas have vestibulocochlear origin^12^; however, about 1%–12% occur in the oral cavity.^3^ Detecting the nerve of origin in the oral cavity is often challenging. Sensory innervation of the posterior third and base of the tongue is performed by the glossopharyngeal and vagus nerves, and motor innervation of the tongue is performed by the hypoglossal and vagus nerves.^13^ It is difficult to say which nerve in our patient originated schwannoma. However, it seems that Schwann cells around the glossopharyngeal, vagus, or hypoglossal nerves were the source of this mass, but the issue is open to discussion. The tongue schwannoma is most commonly seen in the 2nd to fourth decades of life.^9^ Also, our patient was in the early third decade of life (age 21). The choice treatment for tongue schwannoma is surgical excision and the surgery is more complex in those masses located on the base of the tongue.^11^ So far, several surgical techniques have been proposed to remove the tongue schwannoma, the choice depends on the location and size of the mass. Classical techniques include trans‐oral excision, submandibular approach, suprahyoidpharyngotomy, and mandibulotomy with lip splitting.^9^ The trans‐oral surgical excision is the most common and preferred technique.

Two types of schwannoma have been reported in the oral cavity: encapsulated (submucosal type) and non‐encapsulated, located below the mucous membrane's basal stratum.^14^ Most cases of schwannoma are sporadic, but in some subjects, they are associated with neurofibromatosis type II (schwannomatosis).^14^ It has been reported that pain is generally identified in subjects with schwannomatosis rather than sporadic cases, and nerve edema and myxoid alterations have been associated with this condition.^9^ Furthermore, some systematic review studies have reported that about 70% of tongue schwannomas are painless.^10^ Our patient, a case of sporadic schwannoma, did not report any pain in the mouth.

In the present case study, the patient's chief complaint was swelling at the base of the tongue. According to a systematic review study by Sitenga et al. (2017), in 87% of patients with schwannoma of the tongue, the swelling was the most common symptomatic complaint.^10^ According to a study mentioned above, swelling of the base of the tongue is associated with some secondary complications such as dysphagia (46.7%), discomfort (40%), dysarthria (33.3%), snoring (33.3%), nasal breathing (20%), and impaired tongue movement (13.3%). None of the symptoms of dysphagia, dysarthria, difficult nasal breathing, and snoring were seen in this presented patient and only reported an unpleasant sensation behind the tongue. What is certain is that the schwannoma in our patient has not yet reached the stage of obstruction of the posterior airways and the development of snoring, dysphagia, and odynophagia.

## 4 CONCLUSION

Tongue Schwannoma (Neurilemmomas) is rare, and a definite diagnosis is made only with histopathologicalinvestigations. It seems that schwannomas of the tongue base originate from branches of the glossopharyngeal, vagus, or hypoglossal nerves, but the issue is open to discussion. Additionally, Trans‐oral surgical excision is an efficient method to remove most of the schwannoma at the base of the tongue.

## AUTHOR CONTRIBUTIONS

**Mahboobe Asadi:** Data curation; writing – original draft. **Malihe Mohseni:** Writing – original draft. **Fatemeh Jahanshahi:** Supervision; writing – original draft; writing – review and editing. **Arash Esmaeili:** Data curation; writing – original draft. **Zhaleh Mohsenifar:** Data curation; writing – review and editing.

## FUNDING INFORMATION

This study has no financial source and support.

## CONFLICT OF INTEREST STATEMENT

There is no conflict of interest to declare.

## ETHICAL APPROVAL

The present study complies with ethical and research standards involving humans. This article does not contain any studies involving animals performed by any of the authors.

## CONSENT

Written informed consent was obtained from the patient for publication of this case report and the accompanying images. A copy of the written consent is available for review by the editor‐in‐chief of this journal.

## Data Availability

Data in the current study are available from the corresponding author on reasonable request.
